# Methylated Septin9 has moderate diagnostic value in colorectal cancer detection in Chinese population: a multicenter study

**DOI:** 10.1186/s12876-022-02313-x

**Published:** 2022-05-11

**Authors:** Dong-cheng Lu, Qi-fang Zhang, Li Li, Xian-ke Luo, Bin Liang, Yi-han Lu, Bang-li Hu, Hai-xing Jiang

**Affiliations:** 1 Department of Gastroenterology, First Affiliated Hospital of Guangxi Medical University, Shuangyong Road 6, Nanning, 530021 Guangxi China; 2 Department of Gastroenterology, Nanxishan Hospital of Guangxi Zhuang Autonomous Region, Guilin, 541002 China; 3 Department of Gastroenterology, Jiangbin Hospital of Guangxi Zhuang Autonomous Region, Nanning, 530021 China; 4 Department of Gastroenterology, Minzu Hospital of Guangxi Zhuang Autonomous Region, Nanning, 530001 China; 5 Department of Research, Guangxi Medical University Cancer Hospital, Hedi Road 71, Nanning, 530021 China

**Keywords:** Colorectal cancer, Septin9, Methylation, Fecal immunochemical test, Diagnosis

## Abstract

**Background:**

The detection rate of methylated Septin9 (mSEPT9) in colorectal cancer (CRC) is varied greatly across the studies. This study aimed to evaluate the diagnostic ability of mSEPT9 in CRC, and compare the diagnostic efficacy with fecal immunochemical test (FIT).

**Methods:**

326 subjects from four centers were prospectively recruited, including 179 CRC and 147 non-CRC subjects. The plasma was collected for mSEPT9 and CEA, AFP, CA125, CA153 and CA199 test, and fecal samples for FIT tests. Sensitivity, specificity and area under the curve (AUC) of receiver operating characteristic curve were calculated to evaluate the diagnostic value of each biomarker.

**Results:**

The positive rate in mSEPT9 and FIT, and the level of CEA, CA125 and CA199 were significantly higher in CRC compared with non-CRC subjects. The mSEPT9 positive rate was not associated with TNM stage and tumor stage. The sensitivity, specificity and AUC of mSEPT9 in diagnostic CRC were 0.77, 0.88 and 0.82, respectively, while the value in FIT was 0.88, 0.80 and 0.83, respectively. mSEPT9 and FIT have higher AUC value than that of CEA, CA125 and CA199. Combination of both mSEPT9 and FIT positive increased sensitivity and AUC to 0.98 and 0.83, respectively, but the specificity was declined. mSEPT9 has a slightly low sensitivity in diagnosis of colon cancer (0.87) compared with rectal cancer (0.93).

**Conclusion:**

mSEPT9 demonstrated moderate diagnostic value in CRC detection, which was similar to the FIT but superior to the CEA, CA125 and CA199. Combination of mSEPT9 and FIT further improved diagnostic sensitivity in CRC.

*Trial registration*: ChiCTR2000038319.

## Background

Colorectal cancer (CRC) remains one of the most common malignancies in the world and the incidence rate still increased, especially in China [1, 2]. The prognosis of early CRC is greatly improved with the advancement of treatment strategy. However, the prognosis of those CRC patients at advance stage still poor, hence, early detection of CRC becomes a crucial challenge for the clinician. Currently, colonoscopy is the gold standard for CRC detection, but this method is invasive procedure with high risk of complications, which limit its widely application in the clinical setting [3]. Fecal occult blood test (FOBT) or fecal immunochemical test (FIT) is widely used in the clinical setting due to its low cost and non-invasiveness, whereas the sensitivity and specificity of them alone is relatively low and is affect by many confounders [4, 5]. Some tumor biomarkers, such as carcinoembryonic antigen (CEA) and carbohydrate antigen-199 (CA199), are also used to diagnose CRC, but the diagnostic accuracy remain unsatisfactory and they were not recommended for CRC screening in current clinical guideline [6, 7]. Therefore, it is necessary to identify a non-invasiveness method with high diagnostic ability for the detection of CRC.

It well known that DNA methylation is essential for the gene regulation, maintenance of cellular identity and epigenetic changes [8, 9]. Aberrant DNA methylation has been shown to closely link to the pathogenesis of several cancers, including CRC [10–12]. Given that specific methylation of cancer occurs early in tumorigenesis, identifying specific aberrantly methylated genes become attractive strategy for early detection of cancer; in addition, gene methylation appears to be stable, yields an amplifiable signal and can be detected with high diagnostic accuracy [13]. At present, several DNA methylation of genes have been reported to associate with the tumorigenesis of CRC, and some of them could be served as potential screening biomarkers for CRC [14, 15]. Among these biomarkers, methylated Septin9 (mSEPT9) is considered as a promising one for detecting CRC [16].

*SEPT9* gene is located at chromosome 17q25.3 and involves in the process of cytokinesis and cytoskeletal organization [17, 18]. During the CRC carcinogenesis, the promoter region of *SEPT9* gene is hypermethylated and the transcription is compromised [19]. To date, several studies have assessed the diagnostic value of peripheral blood mSEPT9 in the detection of CRC, but the diagnostic accuracy for CRC is differs greatly among each study, in which the sensitivity and specificity varied from 36.6 to 95.6% and 77 to 98.9%, respectively [20–24]. When focus on the Chinese CRC patients, the sensitivity and specificity ranging from 61.2 to 81.9% and 83.6 to 93.7%, respectively [25–27]. The great different diagnostic value might attribute to the low sample size or retrospective design of studies, therefore, whether mSEPT9 is a reliable biomarker for CRC detection in Chinese population remain needs to further determined. In this study, we prospectively detected mSEPT9 degree in the patients who underwent colonoscopy examination, and compared the diagnostic efficacy of mSEPT9 to FIT, which aim to clarify the diagnostic value of mSEPT9 for CRC detection in a Chinese population.

## Materials and methods

### Patients and samples

This hospital-based case–control design study included CRC patients and non-CRC patients. The subjects who underwent colonoscopy examination were prospectively collected from four major hospitals from different cities of Guangxi Province, China. Subjects were recruited from June 2019 and June 2021. This study was approved by the ethics committee of each participating hospital. All the subjects were undertaken with informed consent. This study was registered as at Chinese Clinical Trail (ChiCTR: https://www.chictr.org.cn/listbycreater.aspx, Trial Registration ID: ChiCTR2000038319).

### Sample size estimation

Sample size estimation was based on the following equation: The number of cases (n) = (Uα/δ)^2^(1 − *P*)*P* [28]. The parameters were defined as follows: Uα is set as 1.96, δ is the cut-off value and estimated as 1/5–1/10 of known mSEPT9 sensitivity, and P represented the probability of a positive (putative positive detection rate). According to the equation an estimated 300 cases were required, so in the study a goal was set to collect 300 cases that have complete clinical information.

### Inclusion and exclusion criteria

Inclusion criteria: (1) Subjects who underwent colonoscopy examination were given FIT test and mSEPT9 examinations; (2) The age of subjects ranged from 50 to 75 years old; (3) and fulfills one of the following criteria: the patient has history of intestinal polyp or adenoma; or history of inflammatory bowel disease; or first degree relative history of CRC. Exclusion criteria: (1) Subjects were excluded if they (1) have a history of CRC; (2) intestinal bleeding during the colonoscopy examination; (3) has other cancers or immune diseases or inflammatory diseases.

### Sample collection and storage

A 10 mL peripheral venous blood sample was collected with K_2_EDTA anticoagulant tubes for the mSEPT9 assay kit (BioChain, Beijing, China). Sample storage and transportation followed the instructions for use of the mSEPT9 kit. Sample tests and data analysis followed the manufacturer’s manual.

### SEPT9 methylation detection

The PCR fluorescence probe method was used to detect mSEPT9. First, total cell-free DNA in plasma was extracted from 3.5 mL plasma samples with the use of the plasma processing kit (BioChain). The DNA was incubated with bisulfite. Then, methylated target sequences in the bisulfite converted DNA template are amplified by real-time PCR (RT-PCR). ACTB was used as the internal control. A single 60 µL PCR was performed for each sample in the mSEPT9 assay. The thermal cycling program is as follows: activation at 94 ℃ for 20 min; 45 cycles at 62℃ for 5 s, 55.5 ℃ for 35 s, and 93 ℃ for 30 s; and cooling at 40 ℃ for 5 s. The mSEPT9 was positive if the quantification cycle (“cycle threshold,” Ct) was less than 41 cycles and negative when Ct over 41. RT-PCR was carried out with the 7500 Fast Real time PCR system (Applied Biosystem) using SYBR Green agent (Applied Biosystem).

### FIT assay

FIT is qualitative assay. The stool samples were collected on two consecutive days with the use of the InSure FIT (BioChain) collection card, and the cards were returned to hospitals or central laboratories within 14 days from the first collection. Submitted stool samples were immediately processed and examined using FIT kits (BioChain). FIT assay was conducted before the colonoscopy examination.

### Peripheral blood indicators detection

Routine examination of peripheral blood biomarkers, including hemoglobin, albumin, white blood cell, platelet, neutrophil and lymphocyte were tested using automatic biochemical analyzer. Five tumor biomarkers, including CEA, AFP, CA125, CA153 and CA199 were tested using ECLIA Kit was used for quantitative detection according to the manufacture’s instruction.

### Clinical features collection

The clinical features, including the patients’ age, gender, TNM stage, differentiation, tumor location, were collected. CRC were confirmed by pathological diagnosis. Tumor stages were defined according to TNM staging system of eight edition of the Cancer Staging Manual of American Joint Committee on Cancer [29].

### Statistical analysis

For continuous variables, the differences of between two groups were compared using chi-square test and *t* test where appropriated; and the comparison of over two groups were using one-way ANOVA test. Chi-square test or Fisher's exact test were used to compare the categorical variables where appropriated. For the diagnostic value of mSEPT9 and FIT, sensitivity and specificity with 95% confidence intervals (CIs) were calculated. The receiver operating characteristic (ROC) curve was used to evaluate the diagnostic accuracy, and the comparisons between mSEPT9 and FIT, and the combination were evaluated by the area under the ROC curve (AUC). DeLong's test was used to compared the AUC between two methods. All statistical analyses were performed using the R language (version 4.0.1). Two-side *P*-value < 0.05 was considered statistically significant.

## Results

### Baseline of included participants

The present finally included 326 subjects who underwent colonoscopy examination. There were 211 male subjects and 115 female subjects, with the mean age as 58.6 ± 10.1 years old. Among the subjects, 179 subjects were confirmed colorectal cancer histologically, which were all adenocarcinoma, and the rest 147 subjects were diagnosed as intestinal diseases; the colorectal cancer included 209 colon cancer and 117 rectal cancers, and the non-CRC included polyp, adenoma, inflammation diseases. FIT test showed that 187 patients were positive and 139 negatives, and the mSEPT9 test indicated that 155 patients were positive and 171 negatives.

### Comparison of clinical features in CRC and non-CRC

As the Table 1 showed, there were no greatly difference between CRC and non-CRC subjects in term of mean age, gender, CRC family history, or the complications of diabetes mellitus, hypertension and alcohol (*P* > 0.05), but CRC subjects complicated more hyperlipidemia and smoking than those of non-CRC subjects (*P* < 0.05). Regarding the peripheral blood biomarkers, the value of hemoglobin and albumin was lower in CRC subjects compared with non-CRC subjects, and the number of platelets was obviously increased in CRC patients compared with non-CRC subjects, but no considerably difference between CRC and non-CRC subjects regarding the number of white blood cell, neutrophil and lymphocyte (*P* > 0.05). The levels of tumor biomarkers CEA, CA125 and CA199 were elevated in CRC patients compared with in non-CRC subjects (*P* < 0.05), while the AFP and CA153 has no significant difference between these two kinds of patients (*P* > 0.05). The positive rate of mSEPT9 and FIT were both higher in CRC patients compared with in non-CRC subjects (*P* < 0.05). When compared the difference between patients with polyp and adenoma, we found that adenoma patients with more number smoking status and low-grade of lesion compared with patients with polyp (*P* < 0.05), other indexes showed insignificant difference (*P* > 0.05).

**Table 1:** Comparison of clinical data between CRC and non-CRC subjects

	All	CRC	Non-CRC	*P* value
	N = 326	N = 179	N = 147	
Gender				0.484
Male	211 (64.7%)	113 (62.8%)	98 (67.1%)	
Female	115 (35.3%)	67 (37.2%)	48 (32.9%)	
Age (years)	58.6 ± 10.1	59.5 ± 9.62	57.5 ± 10.5	0.079
Histological type				1.000
No	320 (98.2%)	177 (98.3%)	143 (97.9%)	
Yes	6 (1.84%)	3 (1.67%)	3 (2.05%)	
Diabetes mellitus				0.434
No	303 (92.9%)	165 (91.7%)	138 (94.5%)	
Yes	23 (7.06%)	15 (8.33%)	8 (5.48%)	
Hypertension				0.663
No	252 (77.3%)	137 (76.1%)	115 (78.8%)	
Yes	74 (22.7%)	43 (23.9%)	31 (21.2%)	
Hyperlipidemia				0.012
No	260 (79.8%)	134 (74.4%)	126 (86.3%)	
Yes	66 (20.2%)	46 (25.6%)	20 (13.7%)	
Smoking				< 0.001
No	244 (74.8%)	115 (63.9%)	129 (88.4%)	
Yes	82 (25.2%)	65 (36.1%)	17 (11.6%)	
Alcohol				0.118
No	261 (80.1%)	138 (76.7%)	123 (84.2%)	
Yes	65 (19.9%)	42 (23.3%)	23 (15.8%)	
Location				< 0.001
Colon	209 (64.1%)	90 (50.3%)	119 (81.0%)	
Rectal	117 (35.9%)	89 (49.7%)	28 (19.0%)	
Lesion size (cm)	2.44 ± 1.93	3.13 ± 1.99	1.07 ± 0.67)	< 0.001
Histological type				< 0.001
Adenocarcinoma	179 (54.9%)			
Polyp	97 (29.8%)			
Adenoma	37 (11.3%)			
Inflammation	12 (3.68%)			
Normal	1 (0.31%)			
Hemoglobin (g/L)	125 ± 21.3	119 ± 22.0	135 ± 16.4	< 0.001
Platelet (×10^9^)	273 ± 90.2	296 ± 96.7	242 ± 69.2	< 0.001
Neutrophil (×10^9^)	5.01 ± 12.1	5.68 ± 15.8	4.10 ± 2.01	0.187
lymphocyte (×10^9^)	1.87 ± 0.66	1.88 ± 0.71	1.85 ± 0.60	0.714
Albumin (g/L)	39.4 ± 5.32	37.5 ± 5.04	42.2 ± 4.49	< 0.001
CEA (ng/mL)	48.0 ± 280	78.5 ± 360	3.03 ± 3.64	0.006
AFP (ng/mL)	2.92 ± 2.02	2.97 ± 2.42	2.83 ± 1.24	0.523
CA125 (U/mL)	18.6 ± 50.7	22.8 ± 62.6	11.1 ± 9.15	0.017
CA153 (U/mL)	10.3 ± 6.62	10.2 ± 7.27	10.5 ± 5.36	0.698
CA199 (U/mL)	147 ± 993	239 ± 1275	9.95 ± 9.34	0.018
FIT				< 0.001
Positive	187 (57.4%)	157 (87.7%)	30 (20.4%)	
Negative	139 (42.6%)	22 (12.3%)	117 (79.6%)	
mSEPT9				< 0.001
Positive	155 (47.5%)	137 (76.5%)	18 (12.2%)	
Negative	171 (52.5%)	42 (23.5%)	129 (87.8%)	

*CRC* Colorectal cancer

### Comparison of clinical features in CRC patients with different mSEPT9 positive rate

Table 2 summarized the different clinical features of CRC patients with different mSEPT9 positive rate. Among the 179 CRC patients, 137 patients were mSEPT9 positive (76.5%, 137/179). In order to determine the clinical significance of mSEPT9 in CRC, we compared the mSEPT9 positive rate in each clinical feature of CRC patients. The results showed that no significant difference between the positive rate of mSEPT9 regarding the gender and age of CRC patients (*P* > 0.05). With respect to the peripheral blood biomarkers, only the platelet and CA199 showed significant higher value in mSEPT9 positive patients than to negative patients (*P* > 0.05). The positive rate of mSEPT9 was also has no significant difference regarding the TNM stage and tumor stage (*P* > 0.05). There also was no remarkably difference between mSEPT9 positive and negative patients regarding the FIT positive rate (*P* > 0.05).

**Table 2:** Comparison of mSEPT9 positive rate in clinical features of CRC patients

	Positive	Negative	*P* value
	N = 137	N = 42	
Gender			1.000
Male	86 (62.8%)	26 (61.9%)	
Female	51 (37.2%)	16 (38.1%)	
Age (years)	60.1 ± 9.97	57.4 ± 8.28	0.081
Tumor size (cm)	3.26 ± 2.06	2.61 ± 1.68	0.049
T stage			0.067
NR	2 (1.46%)	3 (7.14%)	
T1	5 (3.65%)	2 (4.76%)	
T2	13 (9.49%)	9 (21.4%)	
T3	92 (67.2%)	23 (54.8%)	
T4	19 (13.9%)	5 (11.9%)	
TX	6 (4.38%)	0 (0.00%)	
N stage			0.066
NR	3 (2.19%)	3 (7.14%)	
N0	45 (32.8%)	21 (50.0%)	
N1	38 (27.7%)	10 (23.8%)	
N2	47 (34.3%)	8 (19.0%)	
NX	4 (2.92%)	0 (0.00%)	
M stage			0.087
NR	2 (1.46%)	3 (7.14%)	
M0	102 (74.5%)	34 (81.0%)	
M1	28 (20.4%)	5 (11.9%)	
Mx	5 (3.65%)	0 (0.00%)	
Tumor stage			0.352
NR	4 (2.92%)	3 (7.14%)	
I	16 (11.7%)	8 (19.0%)	
II	33 (24.1%)	11 (26.2%)	
III	57 (41.6%)	15 (35.7%)	
IV	27 (19.7%)	5 (11.9%)	
Hemoglobin (g/L)	118 ± 22.1	122 ± 21.6	0.239
Platelet (× 10^9^)	305 ± 103	270 ± 66.1	0.011
Neutrophil (× 10^9^)	6.11 ± 18.0	4.28 ± 2.08	0.247
lymphocyte (× 10^9^)	1.89 ± 0.69	1.86 ± 0.79	0.865
Albumin (g/L)	37.4 ± 4.20	37.9 ± 7.16	0.656
CEA (ng/mL)	80.0 ± 351	73.1 ± 395	0.920
AFP (ng/mL)	2.69 ± 1.33	3.92 ± 4.34	0.085
CA125 (U/mL)	21.4 ± 62.3	27.6 ± 64.1	0.590
CA153 (U/mL)	9.98 ± 4.90	11.1 ± 12.4	0.588
CA199 (U/mL)	296 ± 1444	42.4 ± 127	0.045
FIT			0.209
Positive	123 (89.8%)	34 (81.0%)	
Negative	14 (10.2%)	8 (19.0%)	

*NR* Not reported

### Diagnostic value of FIT and mSEPT9 in CRC

**Fig. 1:**
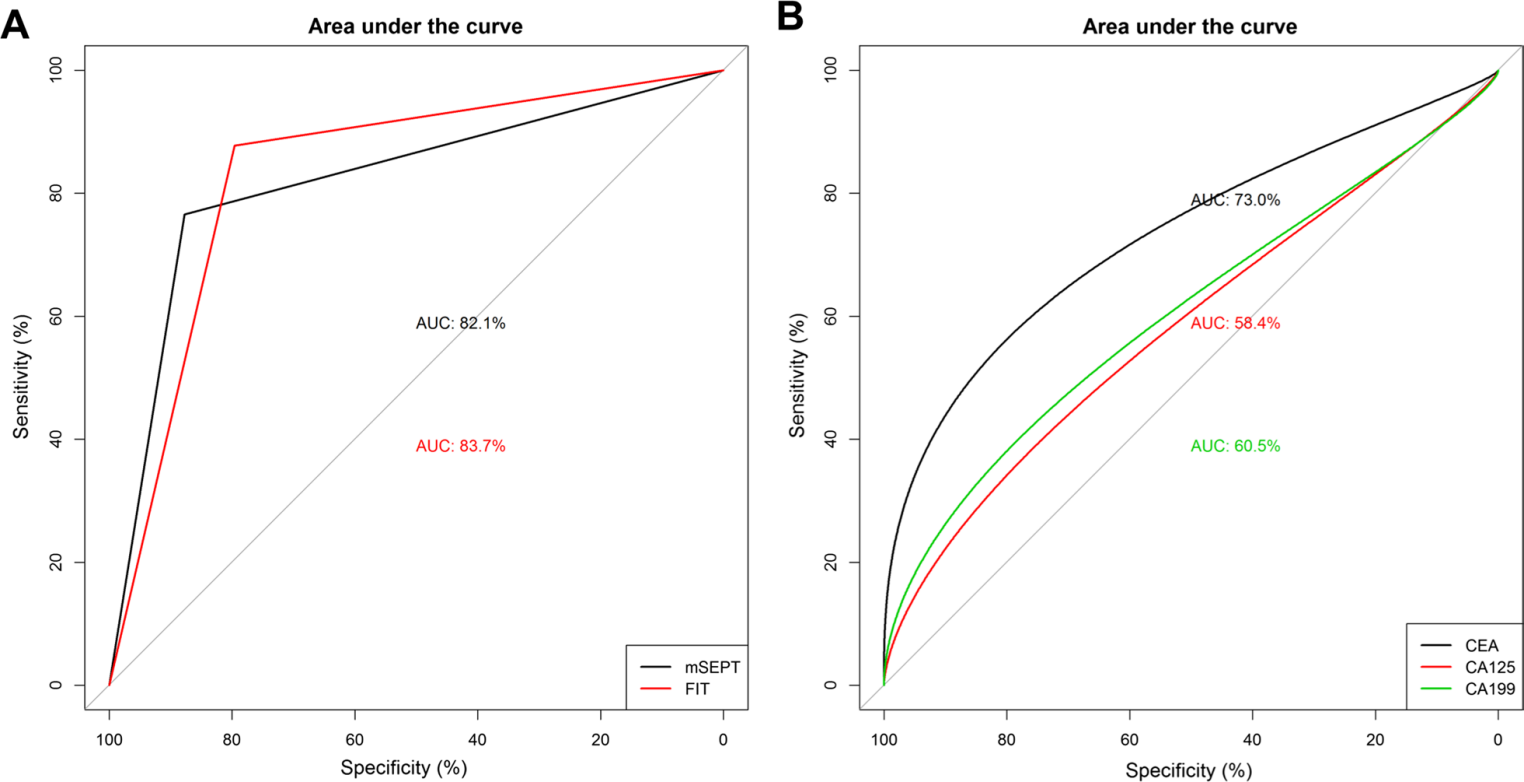
Diagnostic value of mSEPT9, FIT and tumor biomarkers in CRC. **A** Diagnostic value of mSEPT9 and FIT; **B** Diagnostic value of CEA, CA125 and CA199

We applied non-CRC subjects as control and conducted the diagnostic test for mSEPT9 and FIT. As the Table 3 listed, the sensitivity and specificity of mSEPT9 in diagnosis of CRC were 0.77 and 0.88, with the diagnostic accuracy as 0.82, which was lower than those of FIT, with the value as 0.88, 0.80 and 0.84, respectively. We also tested the diagnostic value of CEA, CA125 and CA199 that the levels were increased in CRC, and found that the diagnostic value of these biomarkers was lower, with the AUC as 0.73, 0.58 and 0.60, respectively. There was no significance between mSEPT9 and FIT regarding the diagnostic ability for CRC (*P* = 0.602, Fig. 1). These results suggested that the mSEPT9 has a moderated diagnostic value in CRC, which is similar to that of FIT.

**Table 3:** Diagnostic value of mSEPT9 and FIT in CRC

	mSEPT9	FIT
Sensitivity	0.77 (0.70, 0.83)	0.88 (0.82, 0.92)
Specificity	0.88 (0.81, 0.93)	0.80 (0.72, 0.86)
PPV	0.88 (0.82, 0.93)	0.84 (0.78, 0.89)
NPV	0.75 (0.68, 0.82)	0.84 (0.77, 0.90)
Accuracy	0.82 (0.77, 0.85)	0.84 (0.79, 0.88)
AUC	0.82(0.78, 0.86)	0.83 (0.79, 0.88)

*PPV* Positive predictive value; *NPV* Negative predictive value; *AUC* area under the curve

### Combination of mSEPT9 and FIT in diagnosis of CRC

In order to explore the diagnostic value of the combination of mSEPT9 and FIT, we established two kinds of combination, including Combination I: only both mSEPT9 and FIT positive was considered as CRC; Combination II: either mSEPT9 or FIT positive was considered as CRC. In Combination I, we found that although the overall diagnostic value was similar to Combination II (AUC: 0.83 vs. 0.82), the sensitivity was significantly increased (0.98), but the specificity was decreased, which was only 0.69. In Combination II, the sensitivity specificity was declined to 0.69, but the specificity was increased to 0.96 (Table 4). These results demonstrated that these two kinds of combinations of mSEPT9 and FIT have different diagnostic value in CRC.

**Table 4:** Combination of mSEPT9 and FIT in diagnosis of CRC

	mSEPT9^+^ AND FIT^+^	mSEPT9^+^ OR FIT^+^
Sensitivity	0.98 (0.94, 1.00)	0.69 (0.61, 0.77)
Specificity	0.69 (0.61, 0.75)	0.96 (0.91, 0.98)
PPV	0.72 (0.65, 0.78)	0.93 (0.86, 0.97)
NPV	0.98 (0.93, 1.00)	0.79 (0.73, 0.84)
Accuracy	0.82 (0.77, 0.86)	0.84 (0.79, 0.88)
AUC	0.83(0.79–0.87)	0.82 (0.78, 0.86)

*PPV* Positive predictive value, *NPV* negative predictive value, *AUC* area under the curve

### Diagnostic value of mSEPT9 in colon cancer and rectal cancer

We further determined the diagnostic value of mSEPT9 in different location of CRC. As Table 5 shown, although the overall diagnostic accuracy of mSEPT9 was similar in both colon cancer and rectal cancer, the sensitivity and specificity of mSEPT9 was decreased in colon cancer compared with that in rectal cancer. In contrast to the results of mSEPT9, the sensitivity and specificity of FIT was increased in colon cancer compared with rectal cancer. Collectively, these results indicated that mSEPT9 has a relatively high diagnostic accuracy in colon cancer compared with rectal cancer.

**Table 5:** Diagnostic value of FIT and mSEPT9 in CRC

	mSEPT9		FIT	
	Colon cancer	Rectal cancer	Colon cancer	Rectal cancer
Sensitivity	0.87 (0.79, 0.92)	0.93 (0.76, 0.99)	0.82 (0.74, 0.89)	0.68 (0.48, 0.84)
Specificity	0.74 (0.64, 0.83)	0.79 (0.69, 0.87)	0.81 (0.71, 0.89)	0.94 (0.87, 0.98)
PPV	0.81 (0.73, 0.87)	0.58 (0.42, 0.72)	0.85 (0.77, 0.91)	0.79 (0.58, 0.93)
NPV	0.81 (0.71, 0.89)	0.97 (0.90, 1.00)	0.78 (0.68, 0.86)	0.90 (0.82, 0.95)
Accuracy	0.81(0.75, 0.86)	0.82(0.74, 0.89)	0.82(0.76, 0.87)	0.88(0.81, 0.93)
AUC	0.81(0.75, 0.86)	0.81(0.75, 0.86)	0.82 (0.76, 0.87)	0.81 (0.72, 0.90)

*PPV* Positive predictive value, *NPV* negative predictive value, *AUC* area under the curve

### Diagnostic value of mSEPT9 in CRC at early or advance stage

Finally, we determined the diagnostic value of mSEPT9 in different stage of CRC. We defined patients with tumor stage I and II as early stage, otherwise advance stage, and then calculated the diagnostic value of mSEPT9 at early stage and advance stage. We found that, the sensitivity, specificity and AUC value of mSEPT9 similar between early stage and advance stage, with the AUC value as 0.83 and 0.84, respectively (Table 6), indicating that no significant difference of mSEPT9 diagnostic value regarding different tumor stage.

**Table 6:** Diagnostic value of mSEPT9 in CRC at early or advance stage

	Early stage	Advance stage
Sensitivity	0.80 (0.72, 0.86)	0.80 (0.72, 0.86)
Specificity	0.88 (0.78, 0.94)	0.87 (0.80, 0.93)
PPV	0.93 (0.87, 0.97)	0.89 (0.83, 0.94)
NPV	0.68 (0.58, 0.77)	0.76 (0.68, 0.83)
Accuracy	0.82(0.76, 0.87)	0.83 (0.78, 0.87)
AUC	0.84(0.79, 0.89)	0.83 (0.79, 0.88)

*PPV* Positive predictive value, *NPV* negative predictive value, *AUC* area under the curve

## Discussion

In the present, we prospectively recruited 326 subjects who underwent colonoscopy examination, and tested the mSEPT9 and FIT for each subject. We found that the positive rate of mSEPT9 was significantly increased in CRC compared with non-CRC subjects. We also found that positive rate of mSEPT9 was associated with CEA, CA125 and CA199 levels, but not the age, gender, TNM stage, tumor stage and FIT positive rate. Then we determined the diagnostic value of mSEPT9 on CRC, the results showed that the sensitivity, specificity and the diagnostic accuracy was 0.77, 0.88 and 0.82, respectively. We noted that the diagnostic accuracy of FIT was similar to that of mSEPT9. Next, we determined the combination of mSEPT9 and FIT in diagnosis of CRC, however, the results showed that the sensitivity and specificity was varied greatly between two kinds of combinations. Finally, we found that the specificity of mSEPT9 was similar in both colon cancer and rectal cancer, while the sensitivity was higher in rectal cancer than in colon cancer. Taken together, these results demonstrated that plasma mSEPT9 was associated with the pathogenesis of CRC, and has a moderate diagnostic value on CRC, while the combination of FIT could not significantly increase the diagnostic value on CRC.

In this study, the number of platelets was obviously increased in CRC patients compared with non-CRC subjects, which was similar to the previous reported [30], suggested that CRC patients usually present hypercoagulation status compared with non-CRC patients [31], and also probably used to predict the survival of CRC patients [32]. Since gene methylation occurs in distinct genomic areas and associated with epigenetic transcriptional silencing of tumor suppressor genes, detection of gene methylation in samples from tissue or blood are becoming novel candidate targets for detecting cancer. A recent study has shown that the DNA methylation frequency of *SEPT9* gene up to 91.8% (90/98) in CRC tissues, indicating a high methylation status of *SEPT9* in CRC [33]. In addition, mSEPT9 DNA is found to be released into the peripheral blood from necrotic and apoptotic cancer cells during CRC carcinogenesis; thus, CRC could be determined by testing the degree of mSEPT9 in peripheral blood, which makes it likely to be a promising non-invasive tumor biomarker for CRC detection. There have been several studies confirmed that detecting mSEPT9 in peripheral blood indicates the presence of CRC, with the positive rate range from 72.4 to 77.0% [34–37]. In our studies, the positive rate of plasma mSEPT9 in CRC and non-CRC was 76.5% and 23.5%, respectively, which was within the range of previous reports, suggesting the high positive rate in CRC. To be note, we failed to show the significant difference among tumor stage of CRC regarding mSEPT9 positive rate, suggesting that mSEPT9 might not be a reliable biomarker to detect CRC at early stage.

The diagnostic value of plasma mSEPT9 has been reported in previous studies, but the diagnostic accuracy of mSEPT9 in CRC detection varied greatly among published literatures. Currently, there are mainly three methods to test the degree of mSEPT9, including RT-PCR assay, Epi proColon test 1.0 and Epi proColon test 2.0. There was study showed that no significant differences between Epi proColon 2.0 and PCR fluorescence probe method [37]. Therefore, the disparity of diagnostic value might due to different ethnicity or other factors. For European and American population, the sensitivity ranged from 46.6 to 90.0%; but the sensitivity varied from 69 to 88% in Chinese population [25–27]. More recently, Chen et al. reported that by recruiting 213 individuals including 91 CRC patients, the sensitivity and specificity of plasma mSEPT9 in diagnosis of CRC was 75.8% and 94.7%. However, Lee et al. [21] reported a much lower sensitivity of peripheral blood mSEPT9 (36.6%) in CRC among Korean population. These results suggested that the diagnostic accuracy of might partly due to different ethnicity. Regarding the cost effectiveness of *mSEPT9* assay for CRC detection, the cost of each test of *mSEPT9* assay is average 600 RMB, and the FIT or FOBT is less than 10 RMB, considering the similar diagnostic value between *mSEPT9* and FIT or FOBT, these results did not support the high-cost effectiveness of *mSEPT9* assay for CRC detection.

When compared the diagnostic value of mSEPT9 with other tumor biomarkers (CEA, CA125 and CA199), we found that the mSEPT9 has better diagnostic performance than those tumor biomarkers. Several studies have shown that combination of mSEPT9 with FIT or FOBT could increase the diagnostic value in CRC. There was study reported that the combination of mSEPT9 and FOBT improved sensitivity to 84.1% compared with single mSEPT9 (61.8%) [35]. While Wu et al. [37] showed that the sensitivity of combination of mSEPT9 and FOBT up to 94.2%. In the present study, we found that either consider one of methods positive (mSEPT9 or FIT), or both of the two methods positive as CRC, the sensitivity and specificity could not increase simultaneously. We also analyzed the diagnostic value of mSEPT9 in colon cancer and rectal cancer was similar, suggesting that the location of tumor did not affect the overall diagnostic value. Collectively, these results indicate the combination of the two methods should be used in considering different clinical setting, which is for screening or excluding CRC.

This study has several strengths. First, this study is a prospectively design study with relatively completed clinical information, which reduce the selected bias and the influence of potential confounders. Second, the CRC patients in this study are from multiple centers with a relative larger samples size, which increased the statistical power and guarantee the robustness of diagnostic accuracy. There were several major limitations in this study, although our study recruited a larger sample size of patients, the number in subgroup analysis, such as tumor stage, tumor location, remain small. Thus, further study with large number is warranted to verify the results in subgroup analyses. Second, this study only concerns the diagnostic value of mSEPT9 in CRC detection, the association between mSEPT9 and prognosis of CRC patients still need to investigate in future study.

## Conclusion

The present study found that the positive rate of plasma mSEPT9 was increased in CRC patients, but not associated TNM stage and the tumor stage. mSEPT9 has a moderate diagnostic value in diagnosis of CRC, the combination of both mSEPT9 with FIT positive could improve sensitivity, though the specificity decreased in CRC detection. Plasma mSEPT9 together with FIT test is helpful in screening patients at high-risk of CRC.

## Data Availability

The data used to support the findings of this study are available from the corresponding author upon reasonable request.
